# The Philanthropic Index and Review

**Published:** 1894-12

**Authors:** 


THE PHILANTHROPIC INDEX AND REVIEW. Published monthly by Dr. C. T. Wilbur, Kalamazoo, Michigan. Price, fifty cents a year.

This four-page quarto is devoted to the interests of feeble-minded children. It is published at a select school and home for feeble-minded children in Kalamazoo, which is presided over by Dr. Wilbur, the editor of the journal.

